# Recombinant expressing angiopep-2 fused anti-VEGF single chain Fab (scFab) could cross blood–brain barrier and target glioma

**DOI:** 10.1186/s13568-019-0869-3

**Published:** 2019-10-15

**Authors:** Xuemei Ji, Hongyan Wang, Yue Chen, Junfei Zhou, Yu Liu

**Affiliations:** State Key Laboratory of Natural Medicines, School of Life Science and Technology, China Pharmaceutical University, 639 Longmian Road, Nanjing, 211198 China

**Keywords:** Glioma, Blood–brain barrier, Angiopep-2, ScFab-ANG, Transepithelial permeability

## Abstract

In 2009, the FDA approved bevacizumab for the treatment of adult patients diagnosed with recurrent glioblastoma. However, the poor permeability of the macromolecules across the blood–brain barrier, determined by multifactorial anatomical and physiological milieu, restricts the clinical therapeutic effect of bevacizumab. The low-density lipoprotein receptor related protein 1 (LRP1) is highly expressed in the endothelial cells of the brain capillary and the glioma cells. Angiopep-2 (ANG) is a 19-aa oligopeptide that can bind to LRP1 and penetrate the blood–brain barrier by receptor-mediated transport. Therefore, ANG can be used as a dual-targeting drug delivery carrier into the brain and the glioma sites. In this study, ANG gene was fused with the C-terminal domain of single-chain antigen binding fragment (scFab) of the anti-VEGF antibody and recombinant scFab-ANG protein was expressed and purified using Rosatte (DE3) strain. We confirmed that ANG could carry anti-VEGF-scFab, penetrate a three-dimensional model of the brain tumor, and cross the hCMEC/D3 monolayer in the in vitro blood–brain barrier model. The animal experiments demonstrated that 3 h after the tail intravenous protein injection, the fluorescent signals in the brains of the mice in the scFab-ANG group were stronger than that in the scFab group. Furthermore, the study of the in situ rat glioma model shows that scFab-ANG could target glioma while anti-VEGF-scFab could not. These findings indicate that scFab-ANG had stronger transepithelial permeability and glioma targeting capacity. Thus, it can be a potential candidate drug for glioblastoma therapy.

## Introduction

Glioma is the most common form of primary and malignant brain tumor and is highly aggressive. The global incidence rate for glioblastoma (GBM) is 3.2 per 100,000 people. Only 9.8% of the patients survive in the first 5 years post diagnosis and the median survival time is less than 2 years with approximately 100% relapse rate, even after receiving good treatment (Filley et al. 2017; Lieberman 2017). Surgery alone has limited efficacy for the treatment of aggressive GBM. Currently, postsurgery therapy combined with radiotherapy and chemotherapy is the standard treatment method for newly diagnosed patients with GBM. However, therapeutic drugs are extremely limited due to the intrinsic resistance or multidrug resistance of the malignant glioma cells to chemotherapy drugs (Tanaka et al. 2000). Recently, antibodies have gained prominence for treatment and several antibody-based drugs have been applied for cancer therapy.

Bevacizumab, a recombinant humanized anti-VEGF-A monoclonal antibody (mAb), can block the formation of new blood vessels in tumors and suppress the progression of tumors by reducing the availability of nutrients and oxygen to the surrounding tumor cells (Macovei et al. 2010; Tonra and Hicklin 2007). In 2004, FDA approved it to treat metastatic colorectal cancer in combination with standard chemotherapy. Currently, it is widely used to treat various types of cancers, including metastatic non-small-cell lung cancer (NSCLC), metastatic renal cell carcinoma (RCC), breast cancer, epithelial ovarian cancer, and glioblastoma (Ferrara and Adamis 2016). Anti-VEGF-antibody is widely applied in novel antibody-based drug development, such as anti-VEGF and anti-PD1 bi-specific antibody (Xiong et al. 2018). However, almost all the large-molecule drugs and more than 98% of small-molecule drugs are unable to reach the brain parenchyma due to the presence of the blood–brain barrier (BBB). The BBB is a compact cellular layer found between brain parenchyma and blood capillary network, formed by various cells like the brain microvascular endothelial cells (BMECs), astroglia, basement membrane of capillaries and pericytes. Strong tight junctions are present between these cells which do not allow the transfer of macromolecules via paracellular pathways. For many years, studies have focused on lipid soluble drugs that can penetrate BBB. Based on this, several brain drug delivery platforms have been developed such as liposomes (Dos Santos et al. 2018), nanoparticle-based systems (Cena and Jativa 2018; Vlieghe and Khrestchatisky 2013), cell-penetrating peptides (CPPs) (Skrlj et al. 2013) and stem cells based delivery vehicles (Frank et al. 2009).

The low-density lipoprotein receptor related protein 1 (LRP1) is an endocytic receptor expressed in several tissues including the BBB cells and glioblastoma cells, and is known to mediate ligand transport across the endothelial cell layer of the BBB. Angiopep-2 (ANG) contains 19 amino acids (TFFYGGSRGKRNNFKTEEY) and is derived from the Kunitz domains of aprotinin (Demeule et al. 2008). It has been extensively used to target LRP1 on the BBB and penetrate BBB via receptor-mediated transport. ANG has also been used to modify nanoparticles entrapping chemotherapy drugs or conjugated with small molecule directly (Kim et al. 2018). However, few studies have discussed the positive effects of angiopep-2 on antibody delivery.

In this study, we reformatted a single-chain antigen binding fragment (scFab) of the anti-VEGF antibody by fusing the C-terminal domain of scFab to angiopep-2. The single-stranded structure can avoid incorrect assembly between light chain and Fd region of heavy chain. ScFab has a more stable structure and has a longer half-life than scFv. After induction by IPTG, the fusion protein scFab-ANG was successfully expressed in *E. coli*. The transepithelial permeability of scFab-ANG was verified using multicellular tumor spheroids (MTS) and BBB in vitro and in vivo models. Analysis of the frozen sections of brain tissue, nuclear magnetic resonance assay and living imaging of rats suggested that scFab-ANG possesses the capacity to permeate the BBB and target glioma cells in vivo. Overall, to our knowledge, this is the first report stating that the transportation capacity of the anti-VEGF scFab from blood into brain tissues can be improved by angiopep-2. Hence, the scFab-ANG can be a potential candidate drug for brain diseases such as GBM.

## Materials and methods

### Materials

Dulbecco's Modified Eagle Medium (DMEM)/high glucose was purchased from Gibco corporation, USA. Endothelial Basal Medium (EBM)-2 was from Lonza, USA. Fetal bovine serum (FBS) was obtained from Si Jiqing (Zhejiang, China). Pfu DNA polymerase was purchased from TransGen Biotech (Beijing, China). Various restriction enzymes were purchased from New England Biolabs (Beijing, China). ANG gene was synthesized and pMD18-T-ANG vector was constructed by GenScript Corp (Nanjing, China). T4 DNA ligase and HRP- and FITC-labeled goat anti-human lgG (H + L) secondary antibodies were purchased from Vazyme Biotech (Nanjing, China). Streptococcal protein G agarose beads were purchased from Bestchrom (Shanghai, China). Sulfo-Cyanine 3 maleimide (Cy3) fluorescent dye was purchased from Bioorth Biotech (Nanjing, China). Transwell system was purchased from Corning (NY, USA). Rat-tail collagen type I and FITC-dextran were from Merck KGaA (Darmstadt, Germany). Angiopep-2 gene and primers were synthesized by Genewiz Corporation (Suzhou, China).

### Bacteria strains, cell lines, and animals

DH5α and Rosatte (DE3) strains were cultured in Luria–Bertani (LB) media. The human liver tumor (HepG2), immortalized human cerebral microvascular endothelial (hCMEC/D3), and human astrocytoma (U87 MG) cell lines were purchased from American Type Culture Collection (ATCC). All cell lines were cultured in DMEM supplemented with 10% FBS, 100 U/mL penicillin G, and 100 μg/mL streptomycin sulfate at 37 °C in a humidified atmosphere of 5% carbon dioxide, except the hCMEC/D3 cells that were maintained in DMEM containing 15% FBS. Eight-week-old male ICR mice and Wistar rats were obtained from Qing Longshan Animal Centre (Nanjing, China). All experiments involving mice were carried as per the protocols evaluated and approved by the China Pharmaceutical University.

### Construction of pCold-scFab-ANG and pCold-scFab vectors

The VEGF antibody amino acid sequences of the heavy-chain variable domains (V_H_) and light-chain variable domains (V_L_) was based on the complete human VEGF165 mAb (Liu et al. 2009). The ANG gene originates from the Kunitz domains of aprotinin (Demeule et al. 2008). To obtain the expression vectors for scFab-ANG and scFab, pCold I-PelB-LC vector was first constructed using primers for the Light Chain-F with *Nde*I recognition site and Light chain-R with *Bam*HI recognition sit. The cDNA coding for ANG was amplified from the pMD18-T-ANG vector, by polymerase chain reaction (PCR) using the forward and reverse ANG primers (ANG-F and ANG-R). Then, the gene was linked to the 3′ end of the Fd fragment of the heavy chain by using overlapping PCR to form the fusion gene via a flexible (SG_3_)_2_(SEG_3_)_4_SG_3_SG linker as linker 1, and thus, Fd-ANG was formed. Following this, the gene was digested and ligated downstream of the pCold I-LC vector. The sequence between the anti-VEGF antibody LC fragment and Fd region was (GGGGS)_3_ linker as linker 2. The expression vector pCold I-scFab-ANG was used as a template for scFab-ANG gene cloning and then the scFab-ANG gene was spliced into a new pCold I vector as pCold I-scFab expression vector. The primers and genes sequences are shown in Table 1.

**Table 1:** The primers and gene sequences

	5′ → 3′	Underline
ANG gene	ACCTTCTTTTACGGCGGAAGCAGGGGCAAGAGAAACAACTTCAAGACAGAGGAGTAC	
PelB gene	ATGAAATACCTGCTGCCGACCGCTGCTGCTGGTCTGCTGCTCCTCGCTGCCCAGCCGGCGATGGCC	
Linker 1	TCTGGTGGTGGTTCTGGTGGCGGCTCTGAGGGTGGTGGCTCTGAGGGTGGCGGTTCTGAGGGTGGCGGCTCTGAGGGAGGCGGTTCCGGTGGTGGCTCTGGT	
Linker 2	GGAGGCGGTGGCTCAGGAGGTGGTGGGAGCGGTGGCGGCGGATCC	
Light chain	F: GGAATTCCATATGAAATACCTGCTGCCGACCGCTGCTGCTGGTCTGC	*Nde*I
	R: CGGGATCCGGAGCACTCGGTGGGG	*Bam*HI
Fd of heavy chain	F: GGAATTCTCTGGTGGTGGTTCTGGTGGCGGCTCTGAGGGTGGTGGC TCTGAGGGTGGC	*Eco*RI
	R1: CACCGCTTCCGCCTCCGCCGTGGGTCTTGTCGCAGGACTTG	Overlap
	R2: CCGCTCGAGGTGGGTCTTGTCGCAGGACTTG	*Xho*I
ANG	F: CAAGTCCTGCGACAAGACCCACGGCGGAGGCGGAAGCGGTG	Overlap
	R:GCTCTAGATTATTAGTGGTGGTGGTGGTGGTGATGGTGATGGTGATGATGGT	*Xba*I

### Expression and purification of scFab-angiopep-2 (scFab-ANG)/scFab

The pCold-scFab-ANG and pCold-scFab vector were transformed into Rosatte (DE3) strain. The recombinant strains were cultured in LB media with 100 μg/mL ampicillin and 25 μg/mL chloramphenicol. When the optical density (OD_600_) of *E. coli* was approximately 0.6, scFab-ANG was produced by adding 0.2 mM IPTG and incubating at 16 °C, 150 rpm for 12 h in shaking-flask. After that, 0.5 mM IPTG was used to induce scFab production. The recombinant bacterial cells were collected by centrifugation at 5000 rpm for 10 min; then, bacteria were resuspended with PBS (phosphate buffer saline, KH_2_PO_4_ 42 mM, Na_2_HPO_4_ 48 mM, NaCl 136 mM and KCl 2.6 mM) and lysed using an ultrasonicator. The supernatant was filtered through a 0.4 μm membrane filter and purified by passing through Streptococcal Protein G agarose column according to manufacturer’s instructions. The purity and the molecular weight of purified protein were verified by SDS-PAGE.

### Determination of antigen binding activity of scFab-ANG/scFab

The antigen-binding activities of scFab-ANG and scFab were tested by ELISA. The wells of the ELISA plate conjugated with the VEGF antigen were incubated with scFab-ANG/scFab as primary antibodies overnight at 4 °C. The color reaction was developed by the addition of HRP-labeled secondary antibodies and the colorimetric substrate, 3,3′,5,5′-tetramethylbenzidine (TMB). The EC_50_ values ([Ab’]t and [Ab]t) were determined using a sigmoidal dose–response curve to the data points in GraphPad Prism 5 software, where Y is the absorbance at 450 nm and X is the concentration of the VEGF165 antibody used.

To detect the binding of antibodies with the receptors on the cell surface, HepG2 cells were added into 12-well plates, incubated with scFab-ANG/scFab and subjected to indirect immunofluorescence staining overnight at 37 °C, 5% CO_2_. The HepG2 cells were fixed with 4% paraformaldehyde for 20 min. After blocking, the cells were incubated with scFab-ANG or scFab and then incubated with FITC-labeled secondary antibody.

### ScFab-ANG binding activity of LRP-1 by western blotting

To measure the total protein content of the hCMEC/D3 cells, the cell extract was prepared, separated using 12% SDS-PAGE, and then transferred onto polyvinylidene fluoride (PVDF) membranes using the wet transfer system (Bio-Rad, California, USA). The membrane was blocked with 5% non-fat dry milk diluted with TBST at 37 °C for 2 h and incubated with scFab-ANG and scFab protein in TBST with 3% nonfat milk at 4 °C overnight. Following that, HRP-conjugated mouse anti-human antibody (1:5000) was added, incubated for 2 h at 37 °C and visualized with enhanced chemiluminescence in gel imager (Tanon, Shanghai, China).

### Multicellular tumor spheroids (MTS) permeability assays

To determine the permeability of scFab-ANG into the tumor, we established U87 multicellular tumor spheroids model. We prepared 1.5% agar solution dissolved in PBS and then diluted this solution at a dilution of 1:2 in Minimum Eagle’s Medium (MEM) (was purchased from Gibco corporation) supplemented with 20% FBS. Then, we added 0.5% agar-MEM solution into a 90 mm dish (thickness of 2–3 mm) (Del Duca et al. 2004). U87 cells were seeded onto this 90 mm dish at a density of 2 × 10^6^ cells per dish and cultured in MEM with 20% FBS. The cells formed spherical aggregates on the agar-MEM dish overnight at 37 °C and 5% CO_2_. The medium was changed every 2 days. The regularly shaped compact spheroids were separated on a 96 well plate when their diameter reached approximately 300 μm.

The scFab-ANG and scFab were labeled using Cy3 fluorescent dye in PBS (pH 8.0) with 100 mM NaHCO_3_ (Schneider et al. 2017). Cy3 dye can be conjugated with the sulfhydryl group of scFab-ANG/scFab fragment as the sulfhydryl group is absent in ANG. The reaction was protected from light and incubated for 3 h at 20 °C. After the labeling reaction, the reaction mixture was dialyzed using a mini-dialysis unit (Thermo Scientific, USA) in PBS to remove the free dye. The Cy3-labeled scFab-ANG and scFab were stored at − 20 °C after sterile filtration through 0.22 μm membrane filter under sterile field, away from light. Cy3-labeled scFab-ANG and scFab (a final concentration of 1 mg/mL diluted in MEM contained 20% FBS) were separately added to the wells containing the tumor spheroids and incubated for 20 h at 37 °C and 5% CO_2_.

### Construction of the in vitro BBB model and transepithelial permeability assay

To determine BBB permeability of scFab-ANG, an in vitro BBB model was constructed using the Transwell system. The cell seeding density for hCMEC/D3 was 1.3 × 10^5^ cells/cm^2^ and 0.5 mL of the cell suspension was seeded into the upper chambers of the 12-well tissue culture inserts. The insert was 12 mm in diameter and had a pore size of 0.4 μm. It was coated with 100 μL of rat-tail collagen type I (100 μg/mL) (Eigenmann et al. 2013). The lower chambers were filled with 1.5 mL of complete media. DMEM was replaced by EBM-2 base-media supplemented with 20% FBS after cell seeding. The transendothelial electrical resistance (TEER) (Brayden et al. 2012) of the cellular barriers were measured daily using an epithelial voltammeter (EVOM).

When the TEER values reached the highest potential range, transcytosis assay was performed. Fresh HBSS was added into upper and lower chamber at 37 °C for 30 min and then 0.5 mL of scFab-ANG, scFab, and FITC-dextran (70 kDa) (Srinivasan et al. 2015) were separately added into the upper chamber while fresh HBSS solution was supplemented into the lower chambers. The transwell system was placed at 37 °C again and sampling was done from the lower chamber thrice every 30 min. The apparent permeability coefficient (*P*_*app*_) was calculated using the formula: *P*_*app*_ (cm/s) = ΔQ/(Δt·A·C_0_), transcytosis (J) was calculated as J (μg/cm^2^) = ΔQ/A, where ΔQ is content of samples in the lower chamber, expressed in μg, A is the effective membrane area, Δt is reaction time and C_0_ is the initial concentration of the contents in the upper chamber expressed in μg/mL.

### The in vivo permeability assay for scFab-ANG

The scFab-ANG and scFab molecules were labeled with the Cy3 fluorescent dye (Schneider et al. 2017). The 8-week-old male ICR mice (three mice per group) were treated with Cy3-labeled scFab-ANG or scFab by tail intravenous injection at a dose of 10 mg/kg. After 3 h, cardiac perfusion was performed to flush out the blood. The brain tissue was removed and fixed with liquid nitrogen for a few seconds. The frozen sections of the brain tissue from the ICR mice were prepared and observed using a fluorescence microscope (Skrlj et al. 2013).

### Establishment of an orthotopic C6 glioma rat model and in vivo detection of the scFab-ANG location

An orthotopic brain tumor model was established by implanting C6 glioma cells into rat intracranially (Grobben et al. 2002). Wistar rats were anesthetized with 3% chloral hydrate at a dose of 300 mg/kg by intraperitoneal injection and intracranially administered C6 glioma cells (1 × 10 ^6^ cells) dissolved in 10 μL of serum-free DMEM at a position that was 3 mm right of the sagittal suture, 1 mm rostral of the bregma, and at a depth of 5 mm from the brain surface (initially 6 mm deep, subsequently outwards by 1 mm) at a speed of 1 μL/min, using a brain stereoscope and stereotaxic injector (RWD Biotech Co. Ltd., Shenzhen, China). The behavior of the animals was monitored daily after surgery and the sutures were removed 5 days later. Two weeks after the inoculation, the experimental animals were examined by magnetic resonance imaging (MRI) (Bruker Biospec 7T/20 USR, Bruker, Germany) to observe the growth of the tumor in vivo. The tumor-bearing rats were injected with Cy3-labeled scFab-ANG and scFab individually through tail vein at a dose of 10 mg/kg, while saline was used as control. Then, the rats were monitored using in vivo living imaging system (IVIS Spectrum, PerkinElmer, USA) to observe the optical signals from Cy3 fluorescence (Tsai et al. 2018). Finally, the brains were removed and sectioned for HE staining to verify further.

### Statistical analysis

All values were given as mean ± standard deviation (SD). The different experimental groups were compared using a two-tailed student’s t-test. *p *< 0.05 was considered statistically significant, *p < 0.05, **p < 0.01, ***p < 0.001.

## Results

### Preparation of scFab-ANG and scFab

**Fig. 1:**
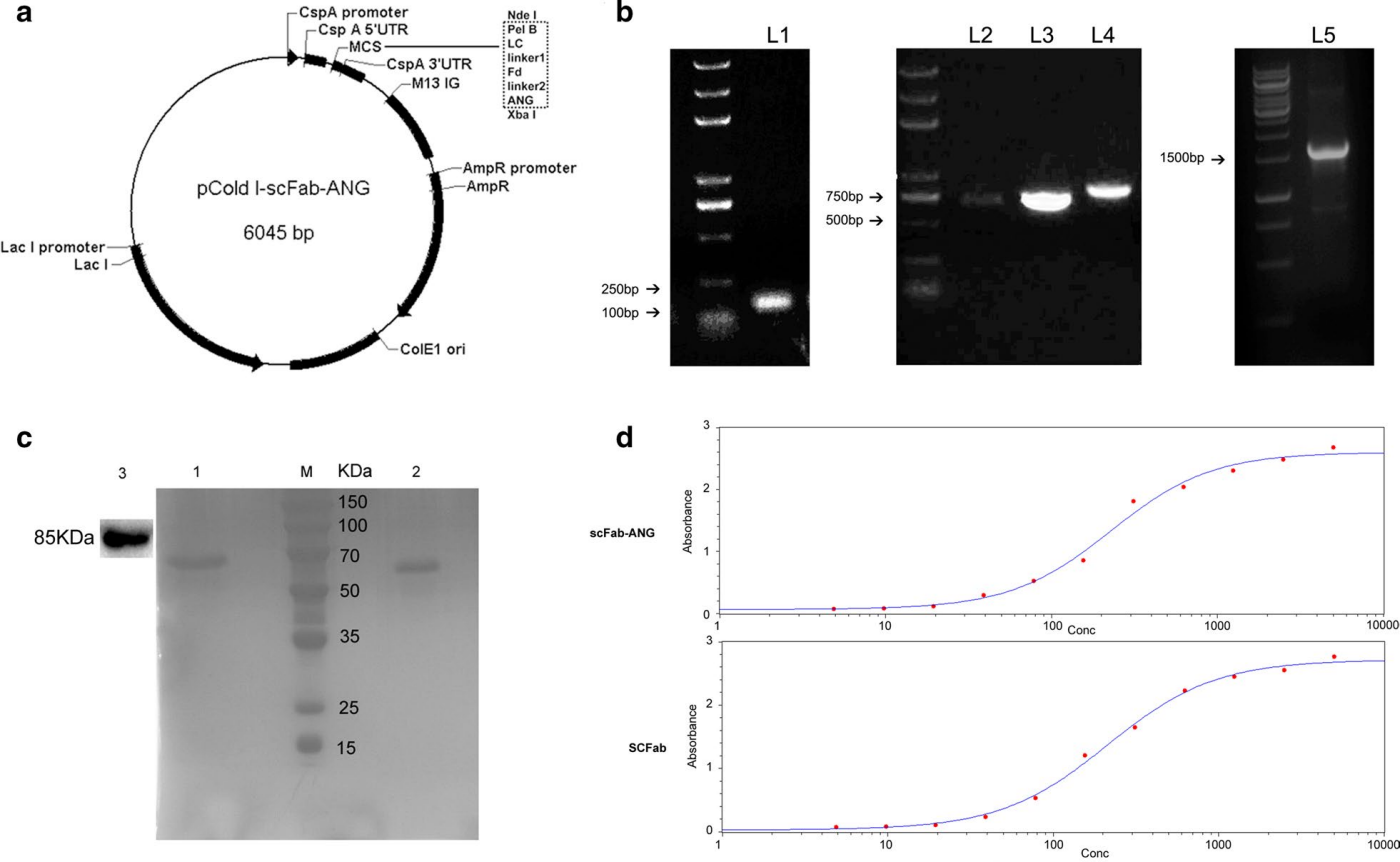
Construction of the scFab-ANG vector and identification of the activity of the fusion protein. **a** Illustration of vector pCold I-scFab-ANG. **b** Agarose gel electrophoresis of nucleic acid amplified by PCR, L1, L2, L3, L4, and L5 correspond to the gene segments of ANG, light chain, Fd domain of heavy chain, Fd-ANG and scFab, respectively. **c** SDS-PAGE gel electrophoresis of scFab-ANG and scFab proteins. Lane 1 is scFab-ANG, Lane 2 is scFab, Lane 3 is western blotting determination using total protein of hCMEC/D3 and LRP-1 molecular weight was about 85 kDa. **d** 4-parameter equation of indirect ELISA of scFab-ANG/scFab

The recombinant scFab-ANG gene, including the relB, Light chain, Fd, and ANG segments, was inserted into the backbone of the expression vector, pCold I (Fig. 1a). To avoid potential interference arising in the protein structures, the genes coding for the light chain, heavy chain, and angiopep-2 were connected using two different linker sequences. The recombinant humanized anti-VEGF antibody antigen binding fragment (Fab) was reformatted as a single-chain Fab (scFab) with a (SG_3_)_2_(SEG_3_)_4_SG_3_SG linker containing 34 amino acids (Hust et al. 2007). ScFab and ANG were fused via a flexible (GGGGS)_3_ linker (Chen et al. 2013). The PelB signal peptide sequence was introduced to enable the translocation of the target protein into the extracellular periplasm. Agarose gel electrophoresis results showed that the size of the gene sequence amplified was equal to the theoretical size (Fig. 1b). The pCold I expression vector system can improve the solubility of recombinant protein due to the presence of the cold shock protein gene promoter. The pCold-scFab and pCold-scFab-ANG vectors were transformed into the Rosetta (DE3) strain, which provided rare tRNA codons to optimize the expression of scFab-ANG and scFab. After induction with 0.2 mM/0.5 mM IPTG for 12 h at 16 °C and 150 rpm, scFab-ANG and scFab were expressed in soluble protein form in the *E. coli* cells, as identified by SDS-PAGE. Then, these cells were ultrasonicated and the supernatant was purified using protein G Sepharose columns. The SDS-PAGE results showed that the molecular weight of scFab-ANG and scFab were approximately 55 kDa, corresponding to the calculated molecular weight (Fig. 1c).

### Antigen affinity activity identification of scFab-ANG and scFab

**Fig. 2:**
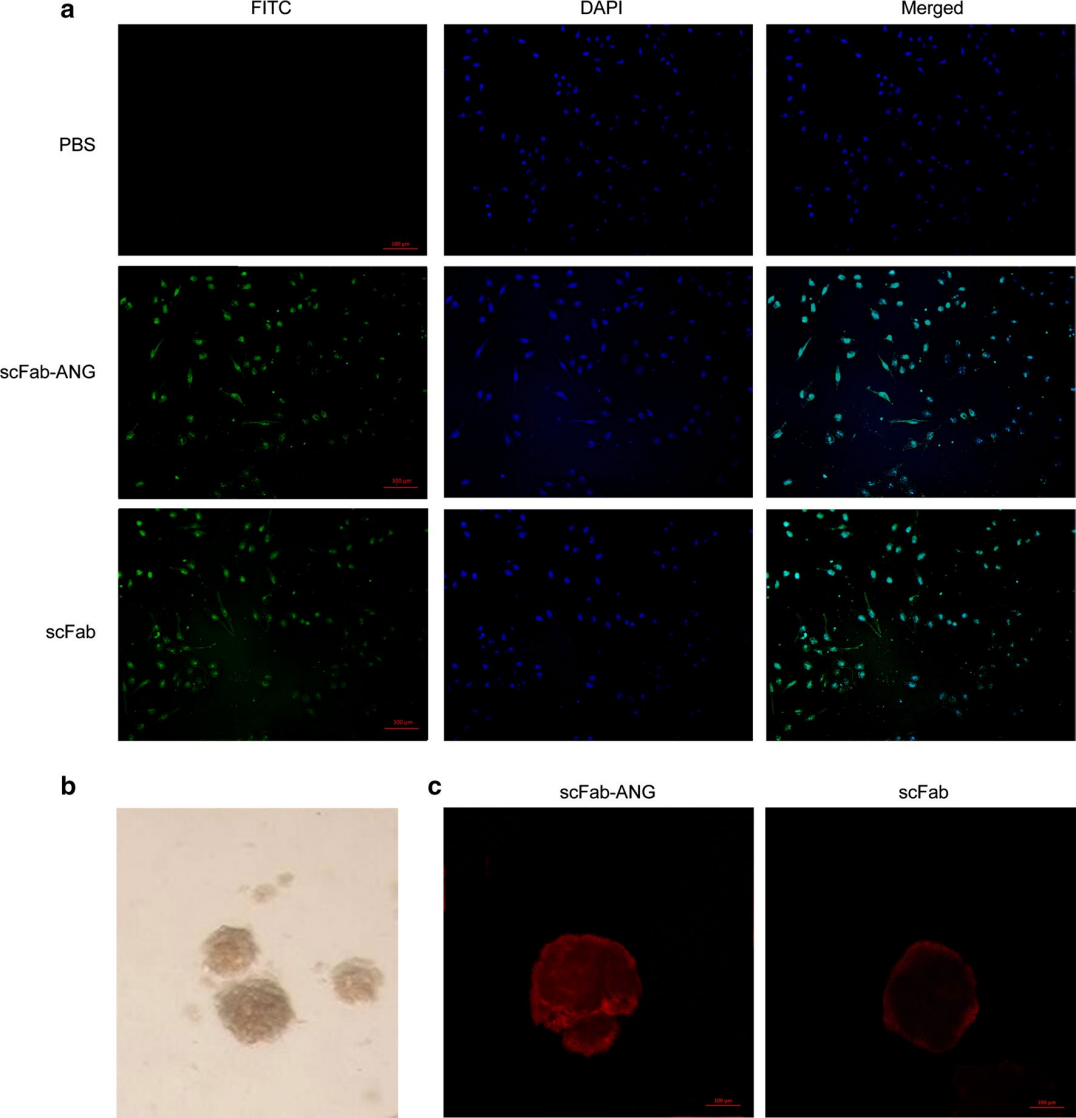
Immunofluorescence assay and multicellular tumor spheroids permeability detection. **a** Fluorescent images of hepG2 cells bound by scFab-ANG and scFab, blue represents nuclei stained by DAPI, green represents binding sites of scFab-ANG/scFab staining by FITC. **b** U87 cells were cultured to form spheroids. **c** Cy3-labeled scFab-ANG (left) and Cy3-labeled scFab alone (right) were separately added to spheroids and cultured for 20 h, spheroids were imaged using confocal microscopy, red represents region stained by Cy3

Sigmoid curves were plotted using the OD values against the antibody concentrations (Fig. 1e). EC_50_ values of scFab-ANG and scFab were 228.19 ng/mL and 216.56 ng/mL, respectively, as determined using indirect ELISA. These results showed there is no significant effect on the binding activity between scFab-ANG and scFab. The LRP-1 binding capacity of scFab-ANG was confirmed by western blotting using goat anti-human IgG antibody, and the molecular weight of LPR-1 was found to be approximately 85 kDa (Fig. 1c, lane 3). While LPR-1 western band couldn't be detected in scFab group. Furthermore, immunofluorescence images showed scFab-ANG and scFab had the ability to bind to the relevant receptors on HepG2 cell surface (Fig. 2a).

### Assessment of MTS permeability of scFab-ANG

To evaluate the permeability of scFab-ANG, a three-dimensional in vitro model of brain tumor was developed. The U87 cells failed to adhere and aggregated to form compact spheroids (Fig. 2b). The diffusion of scFab-ANG and scFab into these spheroids was observed by confocal laser scanning microscope. Fluorescence images showed that the red fluorescent signals corresponding to Cy3-labeled scFab-ANG were significantly stronger than scFab in spheroids. ScFab-ANG was distributed on the surface and penetrated the center of tumor spheroids, while scFab was located at the outer cells of the spheroids (Fig. 2c). The results suggested that ANG was able to greatly increase the tumor permeability of scFab in vitro. Therefore, scFab-ANG may achieve more powerful anti-tumor activity due to the enhanced tumor permeability.

### Assessment of transepithelial permeability of scFab-ANG in vitro BBB model

**Fig. 3:**
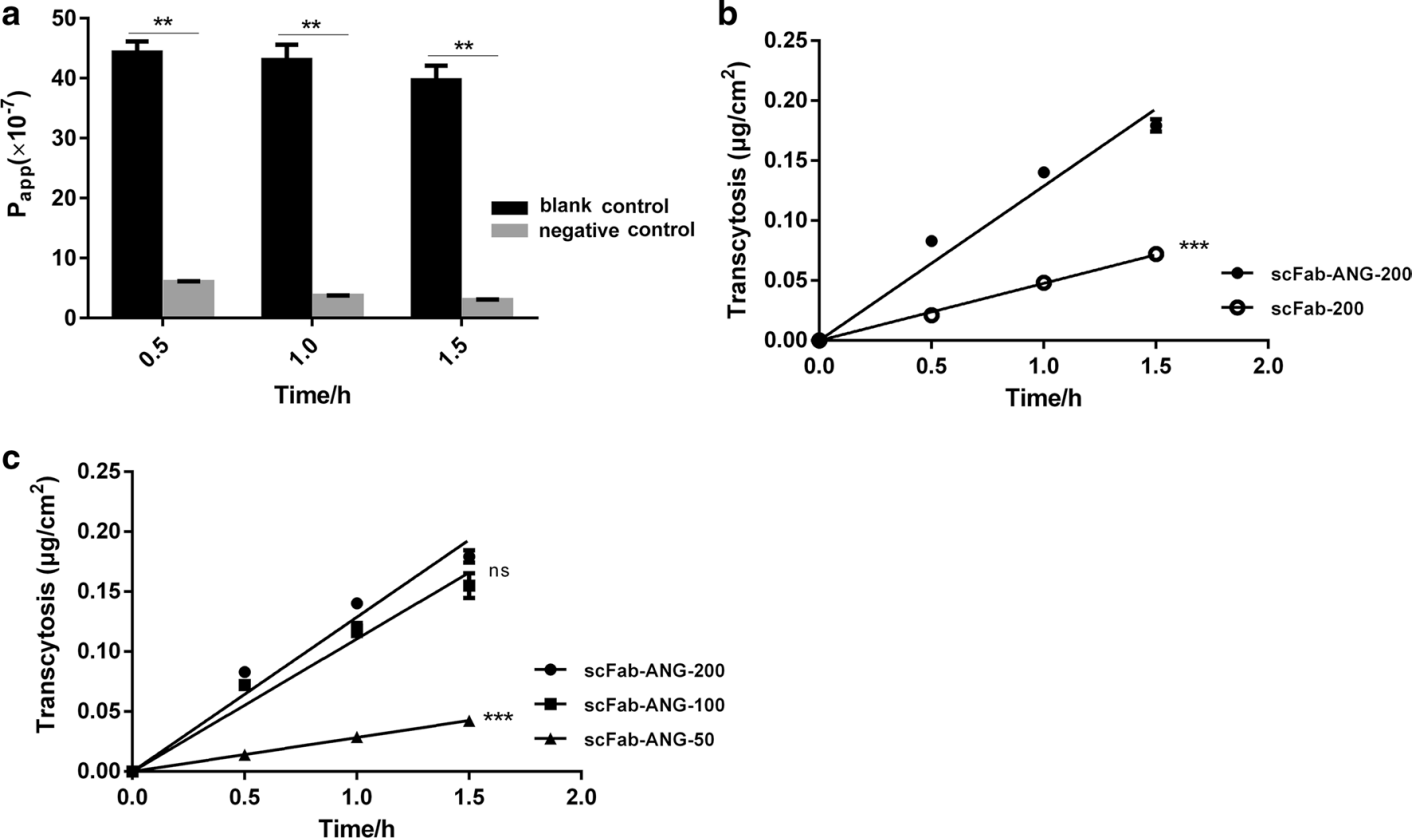
Transport across in vitro BBB model. **a** *P*_*app*_ of FITC-dextran (500 μg) across polycarbonate membrane in negative group (with hCMEC/D3 cell monolayer) or blank control group (without cell monolayer). **b** Transcytosis curve of scFab-ANG and scFab with 200 μg. **c** Transcytosis curve of scFab-ANG (200 μg, 100 μg, 50 μg)

To identify the ability of scFab-ANG to cross the BBB in vitro, we established a BBB model. The integrity of the cellular barriers were evaluated using the TEER values and permeability of FITC-dextran in the inserts with or without cells. We found significant differences in the mean *P*_*app*_ values of FITC-dextran between polycarbonate membrane (without cells) and the hCMEC/D3 cell monolayer. The lower chambers of the inserts without the cells had higher levels of FITC-dextran than the inserts with cells during different time intervals (Fig. 3a). The connections between hCMEC/D3 cells were close enough to be used for assessing the in vitro transepithelial permeability of scFab-ANG and scFab.

When the concentration of the recombinant proteins in upper chambers was 200 μg/mL, the ability of scFab-ANG to permeate the hCMEC/D3 cell layer was more significant than scFab (Fig. 3b), with the transcytosis rate of scFab-ANG being about 2.7 times more than that of scFab (Table 2). ANG could dramatically improve the permeability of scFab in vitro BBB model. ANG enabled the cellular uptake of other drugs and cells with LRP1 with equal efficiency (Mei et al. 2014; Yainoy et al. 2014). There was an increase of transcytosis rate with the initial concentration increase of scFab-ANG in the upper chamber. Transcytosis rate increased by 2.91 times, when the initial concentration was increased from 50 to 100 μg/mL. However, we found the speed increment of transcytosis rates gradually diminished. When the concentration rose to 200 μg/mL, the transcytosis rate rose only by 16.2% compared to that with 100 μg/mL (Table 2 and Fig. 3c). We deduced that LRP1 receptor could be saturated by scFab-ANG, thus, limiting the transport efficiency, which was consistent with previous results (Demeule et al. 2008). These results also verified that the transport of scFab-ANG was mediated by LRP1 on the cell surface.

**Table 2:** Transcytosis rate of scFab-ANG and scFab

Protein	Concentration (μg/mL)	Transcytosis rate (μg/cm^2^/h)
	200	0.129 ± 0.0042
scFab-ANG	100	0.111 ± 0.0038
	50	0.0284 ± 0.00056
scFab	200	0.0477 ± 0.00068

The concentrations of scFab-ANG and scFab in lower chambers were measured every 30 min. Transcytosis curve was generated, X-axis was time, Y-axis was transcytosis. Transcytosis rate was calculated as curve slope

### In vivo assessment of the permeability of scFab-ANG

**Fig. 4:**
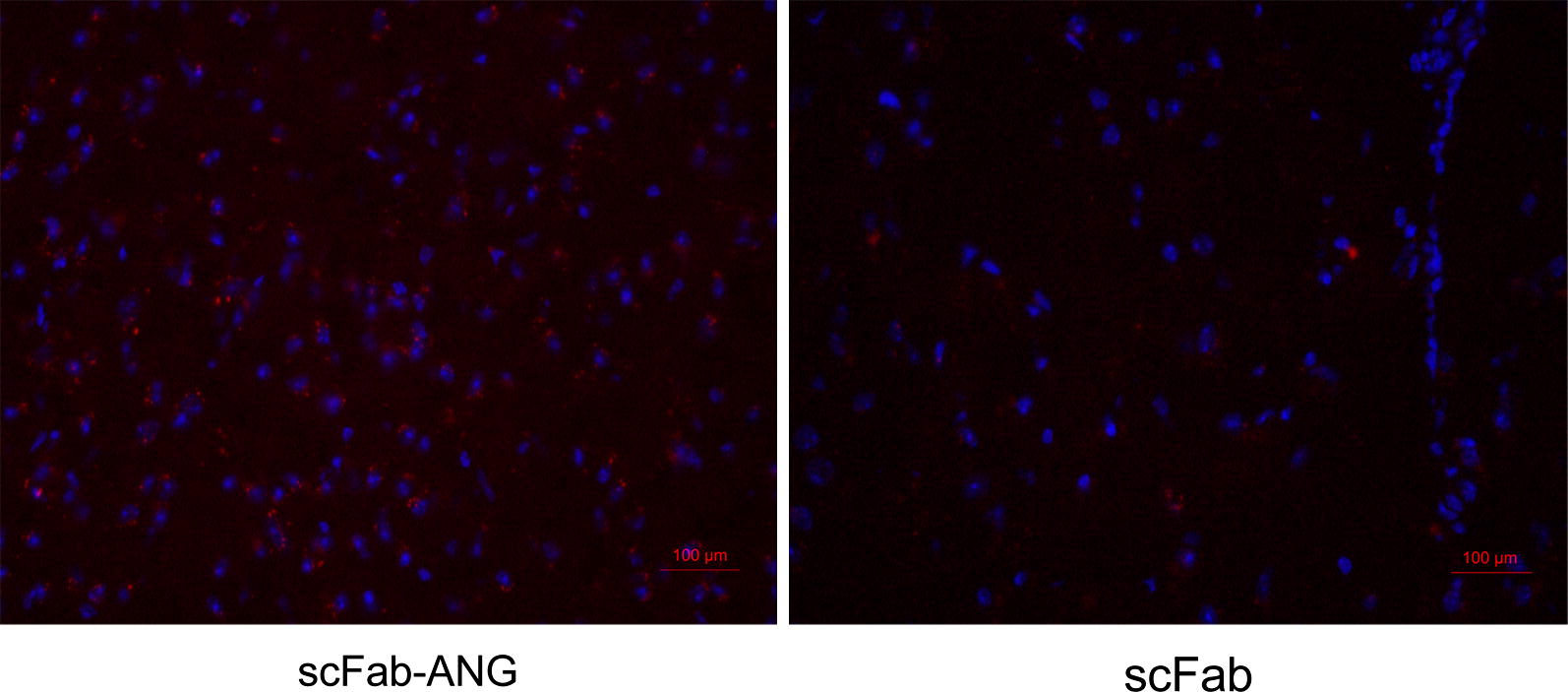
Evaluation of penetration in vivo BBB. Fluorescent images of brain tissue slices in different groups. Blue represents cell nucleus stained by DAPI, red represents Cy3-labeled scFab-ANG (left) and Cy3-labeled scFab (right)

The permeability of scFab-ANG was evaluated using the value showing its accumulation in the brain, in vivo. We selected 8-week-old male ICR mice with well-developed BBB. The merged images showed that the fluorescent signals of scFab-ANG group was stronger than the scFab group (Fig. 4). The unmodified scFab has poor transepithelial permeability and it is scantily distributed in the brain parenchymal. The results demonstrated that the ANG-mediated transcytosis contributed to the accumulation of scFab-ANG in the brain tissues of mice.

### scFab-ANG target glioma in C6 rat glioma model

**Fig. 5:**
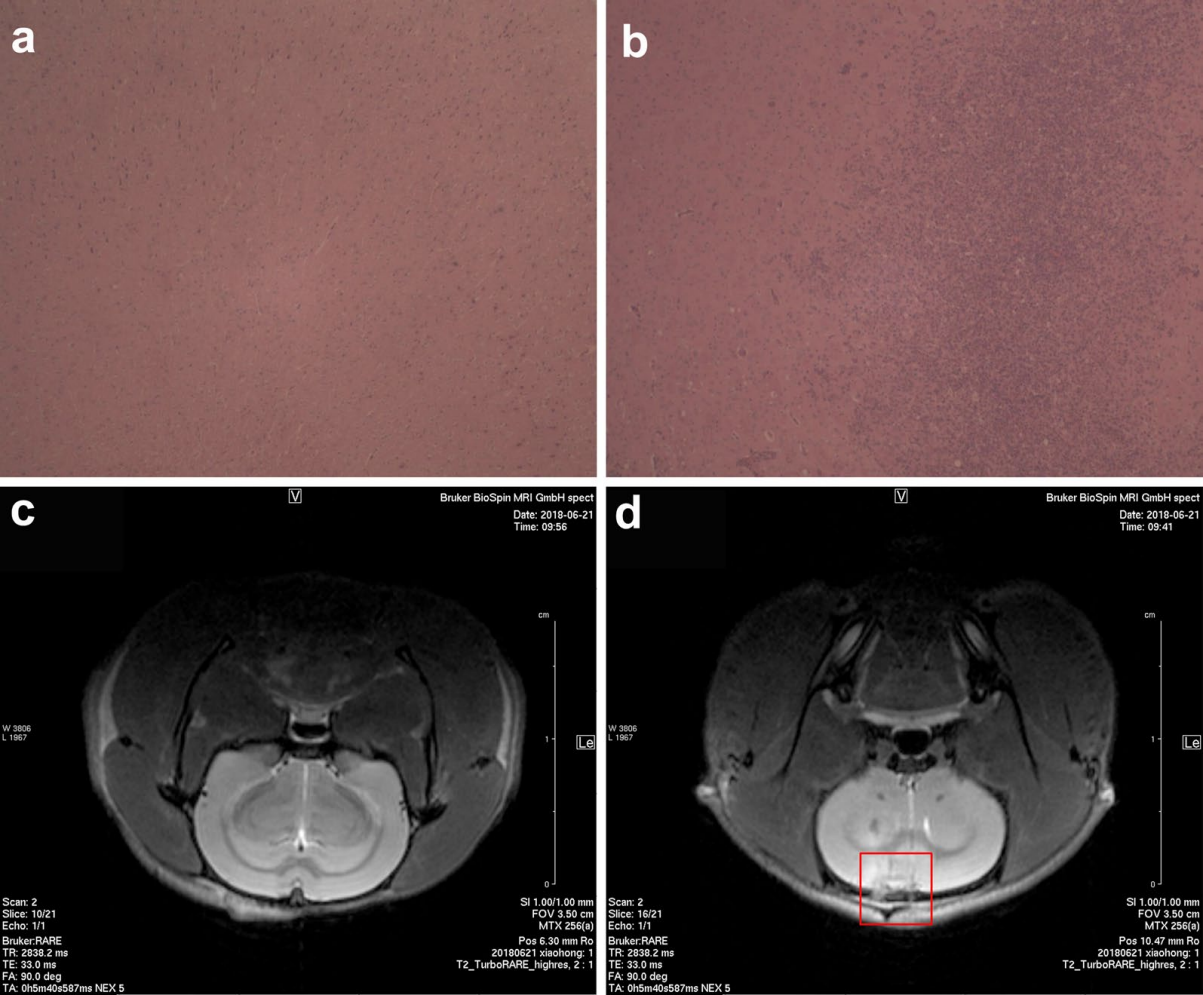
scFab-ANG targets glioma cells in the C6 rat glioma in situ model. HE staining (**a**, **b**) and magnetic resonance imaging (**c**, **d**) were applied for assessing whether C6 glioma cells were successfully inoculated into Wistar rats. **a** and **c** represent the images of rats’ brains that were not inoculated with C6 glioma cells and **b**, **d** represent images of rats’ brains inoculated with C6 glioma cells. The rectangle in **d** represents the site of glioma cells inoculated in the brain

**Fig. 6:**
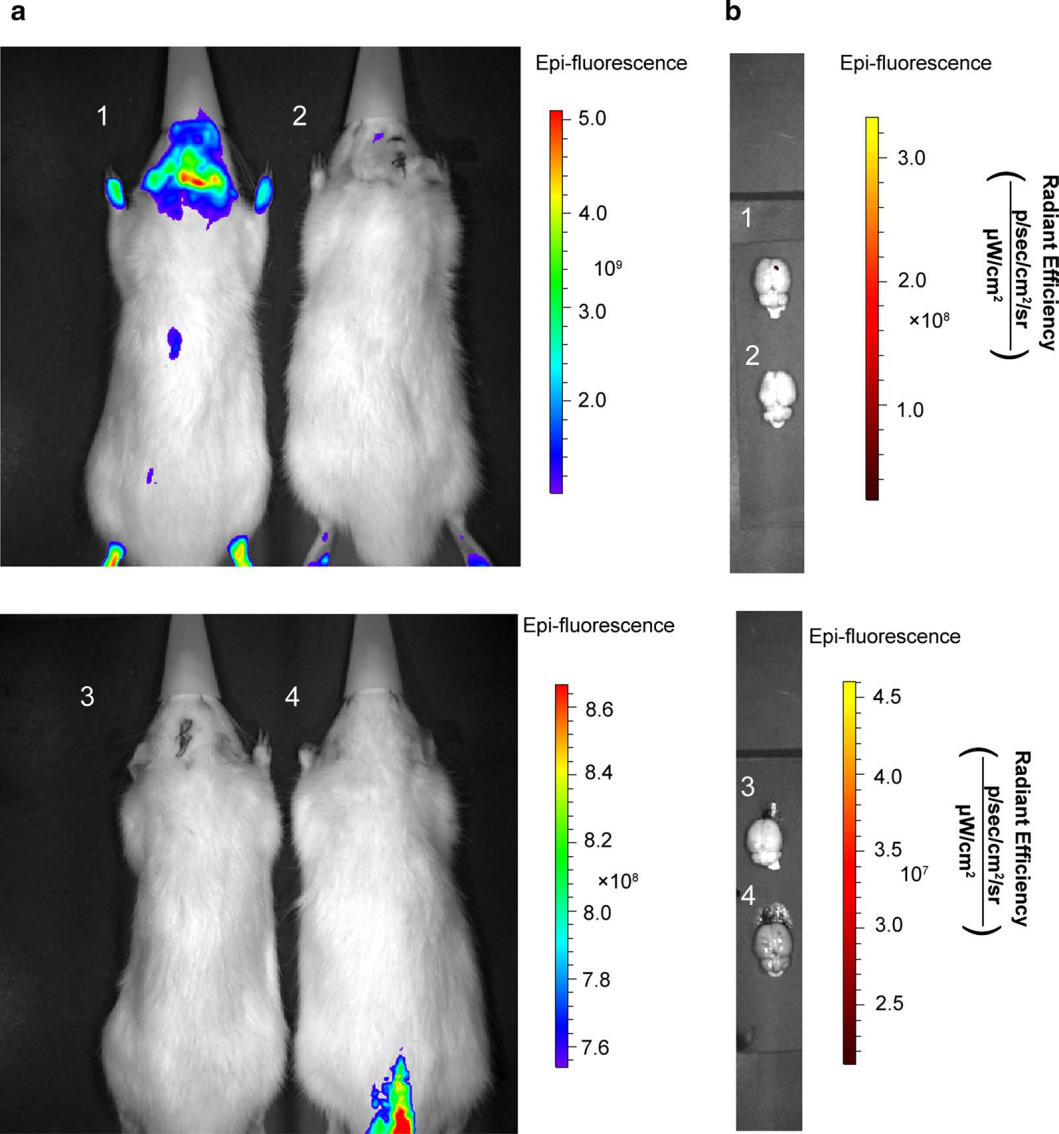
**a** In vivo fluorescence images of glioma-bearing mice intravenously receiving different protein. 1-rat with C6 glioma cells was injected with scFab-ANG; 2-rat with C6 glioma cells was injected with scFab; 3-rat with C6 glioma tumor was injected with saline; 4-rat without C6 glioma cells was injected with scFab-ANG via the tail vein. **b** Ex vivo fluorescence images of brains

To further determine whether scFab-ANG could target glioma in vivo, a rat glioma model was established. Representative HE-stained brain slices images showed that the C6 glioma cells were successfully inoculated into the brains of Wistar rats (Fig. 5a, b). Consistent with the HE assessment, obvious infiltration of tumor cells between the tumor tissues and normal tissues could be seen in the MRI (Fig. 5c, d). Before demonstrating the in vivo therapeutic efficacy, the scFab-ANG were examined for tumor-targeting in Wistar rat bearing orthotopic brain tumors by IVIS. The images in Fig. 6a illustrated the considerable fluorescence signals in the rat’s brain post intravenous administration of scFab-ANG. The orthotopic tumors from the scFab-ANG group exhibited much higher fluorescence signals compared to those treated with scFab or saline apparently because of the targeting effect of angiopep-2. The ex vivo fluorescence signals of brain also illustrated fluorescence signals at tumor sites in the rat post intravenous administration of scFab-ANG (Fig. 6b). The results clearly indicated the significantly increased accumulation of scFab-ANG in the brain tumor.

## Discussion

In recent times, there is a lack of drug delivery systems targeting the brain and hence, it is difficult to deliver large-molecule drugs into the brain, which is protected by the BBB. Several studies investigated methods of breaking the barriers by physical means, such as ultrasound therapies (Meng et al. 2017). However, these methods are risky and unsafe. A “Trojan horse” method allows the passage of biomolecules through the BBB by receptor-mediated transcytosis (RMT). This is the main mechanism used to deliver molecules such as hormones or high molecular mass proteins such as insulin, leptin, low density lipoproteins, and transferrin across the BBB into the brain endothelial cells. Angiopep-2 (ANG) is a ligand of LRP-1 and high levels of LRP-1 expression in BMECs indicate that the drugs modified with ANG will easily pass through the BBB by LPR1-mediated transcytosis. Previous studies have found that scFab has advantages over Fab (Hust et al. 2007). It is easier to assemble the scFab by expressing light chain and Fd fragment of heavy chain than the Fab fragment. The scFab is able to form a stable structure with longer half-life time and does not readily aggregate compared to scFv (Kwong and Rader 2009).

Here, MTS model was established to study the permeability of scFab-ANG. The MTS model simulates some characteristics of tumor microenvironments which are not presented in the cell monolayer, where the spheroid cells are arranged closely (Yuhas et al. 1977). The permeability of the tumor spheroids can better reflect the efficacy of the drug delivery system than the uptake and transport through the cell monolayer. In the in vitro MTS study, the scFab-ANG penetrated the center of the U87 cell tumor spheroids when scFab was distributed in outer cells of spheroids. The result suggested that anti-VEGF scFab-ANG may achieve more powerful anti-tumor activity by inhibiting the formation of blood vessels in the solid tumor. Reducing the tumor blood vessels may slow or stop cancer progression by diminishing the supply of life-sustaining nutrients and oxygen from the blood into the tumor tissues. Using the in vitro BBB model, we found that the increase of transcytosis rate was gradually reduced with increase in the concentration of scFab-ANG in the upper chambers of the inserts. This might be due to the fact that the LRP1 receptors were saturated. However, further studies on this phenomenon are necessary to clarify this. To break the limits, two-ligand modifications of ANG and TAT is advantageous. TAT, independent of the receptor mediated transport, is a positively charged cell-penetrating peptide that improves the cell permeability by interacting with negative charge on the surface of cells, and there is no saturation effect (Fonseca et al. 2009; Torchilin et al. 2001). The results of the in vitro study were consistent with the results of the in vivo study, which showed that much more scFab-ANG was accumulated in the brain parenchyma of the mice than scFab. Based on our results, as a safer and more efficient carrier, ANG can be used for transporting antibody drugs by penetrating BBB using receptor-mediated transport system. Following this, we established an in situ glioma rat model. We detected the distribution of scFab-ANG/scFab in normal brain tissues or tumor sites. The results showed that scFab-ANG could cross the blood–brain barrier and target glioma in vivo.

In this study, we successfully expressed scFab-ANG and scFab using a prokaryotic system. The biological activities of the purified proteins have been proved by in vitro and in vivo experiments. These results suggested that the permeability of scFab was significantly improved by C-terminal ANG and could effectively accumulate in the brain parenchyma of mice and target glioma in in stu model. In addition to the treatment of glioma, ANG can be used to deliver various antibodies to the brain and can be expected to be used for the treatment of many other diseases of brain and central nervous system, such as Alzheimer’s disease. We will perform further studies to extend the application of antibodies and provide more effective antibody-based medicines.

## Data Availability

All data obtained have been included into the manuscript.

## Abbreviations

ANG: angiopep-2
BBB: blood–brain barrier
Cy3: sulfo-cyanine 3 maleimide
DAPI: 4′,6-diamidino-2-phenylindole
DMEM: dulbecco's modified eagle medium
EBM-2: endothelial basal medium-2
ECL: enhanced chemiluminescence
ELISA: enzyme-linked immunoassa
FBS: fetal bovine serum
Fc: crystallizable fragment
FITC: fluorescein isothiocyanate isomer I
GBM: glioblastoma
hCMEC/D3: human cerebral microvascular endothelial cell line
HepG2: liver hepatocellular carcinogen
HRP: horseradish peroxidase
IPTG: isopropyl β-d-thiogalactoside
LRP1: low density lipoprotein receptor related protein-1
MEM: minimum Eagle’s medium
